# Targeted Disruption of Mouse Dip2B Leads to Abnormal Lung Development and Prenatal Lethality

**DOI:** 10.3390/ijms21218223

**Published:** 2020-11-03

**Authors:** Rajiv Kumar Sah, Jun Ma, Fatoumata Binta Bah, Zhenkai Xing, Salah Adlat, Zin Ma Oo, Yajun Wang, Noor Bahadar, Ameer Ali Bohio, Farooq Hayel Nagi, Xuechao Feng, Luqing Zhang, Yaowu Zheng

**Affiliations:** 1 Key Laboratory of Molecular Epigenetics, Institute of Genetics and Cytology, Northeast Normal University, Changchun 130024, China; kum235@nenu.edu.cn (R.K.S.); cissehbah@yahoo.fr (F.B.B.); xingzk444@nenu.edu.cn (Z.X.); land788@nenu.edu.cn (S.A.); zinmaroo23@yahoo.com (Z.M.O.); wangyj559@nenu.edu.cn (Y.W.); noorizever@yahoo.com (N.B.); gene_ali2016@hotmail.com (A.A.B.); haiy@nenu.edu.cn (F.H.N.); zhangluqing@gmail.com (L.Z.); 2 Wenzhou Institute, University of Chinese Academy of Sciences, Wenzhou 325001, China; junma@ciac.ac.cn; 3 Cardiovascular Research Institute, University of California, San Francisco, CA 94158, USA

**Keywords:** Dip2b, LacZ expression, growth restriction, fetal lung development, prenatal lethality, RNA sequencing, mice

## Abstract

Molecular and anatomical functions of mammalian Dip2 family members (Dip2A, Dip2B and Dip2C) during organogenesis are largely unknown. Here, we explored the indispensable role of Dip2B in mouse lung development. Using a LacZ reporter, we explored Dip2B expression during embryogenesis. This study shows that Dip2B expression is widely distributed in various neuronal, myocardial, endothelial, and epithelial cell types during embryogenesis. Target disruption of *Dip2b* leads to intrauterine growth restriction, defective lung formation and perinatal mortality. Dip2B is crucial for late lung maturation rather than early-branching morphogenesis. The morphological analysis shows that *Dip2b* loss leads to disrupted air sac formation, interstitium septation and increased cellularity. In BrdU incorporation assay, it is shown that *Dip2b* loss results in increased cell proliferation at the saccular stage of lung development. RNA-seq analysis reveals that 1431 genes are affected in *Dip2b* deficient lungs at E18.5 gestation age. Gene ontology analysis indicates cell cycle-related genes are upregulated and immune system related genes are downregulated. KEGG analysis identifies oxidative phosphorylation as the most overrepresented pathways along with the G2/M phase transition pathway. Loss of *Dip2b* de-represses the expression of alveolar type I and type II molecular markers. Altogether, the study demonstrates an important role of Dip2B in lung maturation and survival.

## 1. Introduction

There are three Disco-interacting protein 2 (*DIP2*) genes in mammals (*Dip2a*, *Dip2b* and *Dip2c*) and a single *Dip2* gene in *Drosophila melanogaster*, *Caenorhabditis elegans*, bacteria, or single-cell eukaryotes. *Dip2* genes are highly conserved from insects to humans but their biologic functions are still not clarified. Bioinformatic analysis of Dip2 homologs has suggested that DIP2 shares highly conserved domains of DNA methyltransferase-associated protein 1 (DMAP1), Acyl-coA synthetase (AMP-forming; Caic) and AMP-binding site 1 [1]. *Dip2* was initially cloned and characterized in *Drosophila* and mouse using yeast two-hybrid techniques [2]. Since then, Dip2 protein has been extensively studied in *Drosophila*. Studies have shown that *Drosophila Dip2* regulates bifurcation of mushroom body axons and directs axon projection under the regulation of c-Jun N-terminal kinase [3,4]. In *Caenorhabditis elegans*, *Dip2* maintains neuron morphology and axon growth possibly through the AFD domain [5]. In mouse, DIP2 is highly expressed in the brain and may play a role in axon patterning in the central nervous system [2]. In mammals, Dip2A is the most studied family member and is a potential cell membrane receptor of *Fslt1* [6]. Dip2A activation mediates Fstl1 protective effects against neuron and cardiac ischemia apoptosis [6,7]. Similarly, Dip2A/MGMT signaling is important for *Fslt1* regulation in temozolomide resistance in glioblastoma [8]. Furthermore, blocking of the *Fstl1-Dip2a* axis improves anti-tumor immunity [9]. Dip2A knockout blocks acetylation of cortactin and leads to an autism-like phenotype [10]. Besides, *Dip2a* gene is a candidate for developmental dyslexia and autism [11,12].

In contrast to DIP2A protein, little work has been conducted on DIP2B and DIP2C. *Dip2c* gene has been implicated in development delay [13]. Loss of *Dip2c* initiates substantial DNA methylation and epithelial-mesenchymal transition in cancer cells [14]. DIP2B, a family member of DIP2 proteins coded by the *Dip2b* gene, is associated with the fragile site FRA12A on chromosome 12q13.1 in humans [15]. Exosomal transport of mir-133b-3p from mesenchyme to epithelium decreases expression of Dip2B protein and might act as an epigenetic regulator of genes responsible for KIT^+^ progenitor expansion during organogenesis [16]. Defect in DIP2B was also discovered in several bioinformatics studies on disease models including schizophrenia, coronary artery disease (CAD), cervical squamous cell carcinoma, and colorectal cancer [17,18,19,20]. Although these bioinformatics analyses stated a potential intervention of Dip2B in physiopathological processes, the biological role of Dip2B is still far from clear.

The presence of three *Dip2* genes in mammals with possibly distinctive and overlapping functions makes it a challenge to identify in vivo targets of individual DIP2 proteins and their roles in development. To extend the findings, we previously generated *Dip2a* knockout and *Dip2a*-*LacZ* knock-in mice via Crispr-Cas9 technology and showed that *Dip2a* is highly expressed in both neuronal and non-neuronal cell [21,22]. Disruption of *Dip2a* induced spine morphogenesis defect along with thin postsynaptic density and reduced synaptic transmission of pyramidal neurons [10]. Dip2A have a functional role in acetyl-coenzyme A (acetyl-CoA) synthesis [23]. We have also studied the transcriptome of E18.5 embryonic lung and brain under *Dip2a* regulation [24].

In the present study, we demonstrate the biological function of the *Dip2b* gene during embryonic lung development by utilizing a *Dip2b^tm1a^* knock-in mouse model, which was originally generated by KOMP. In the current investigation, the expression pattern of the LacZ gene during embryogenesis was also examined, which is inserted in the *Dip2b* gene during the process of producing *Dip2b* deficient mice. Ablation of the *Dip2b* gene results in growth restriction, significant low birth weight and grossly normal development of offspring. These newborn mice die within a few hours after birth possibly due to obvious neonatal respiratory dysfunction. As for indicators of the adequacy of lung development, we compared *Dip2b^tm1a/tm1a^* newborn with wild type littermates for the presence of respiratory distress, viability, and lung wet:dry ratio. We then performed anatomical and histological analysis of *Dip2b^tm1a/tm1a^* lung from the early stages of embryonic development till birth. In late gestation (E18.5), we evaluated cell proliferation, cell death, collagen deposition, differentially expressed genes, and mRNA level of markers for alveolar and bronchiolar cell subtypes. Our findings depict the biological role of *Dip2b* in perinatal lung maturation and animal survival.

## 2. Results

### 2.1. Dip2b Is Expressed in Multiple Organs during Development

**Figure 1:**
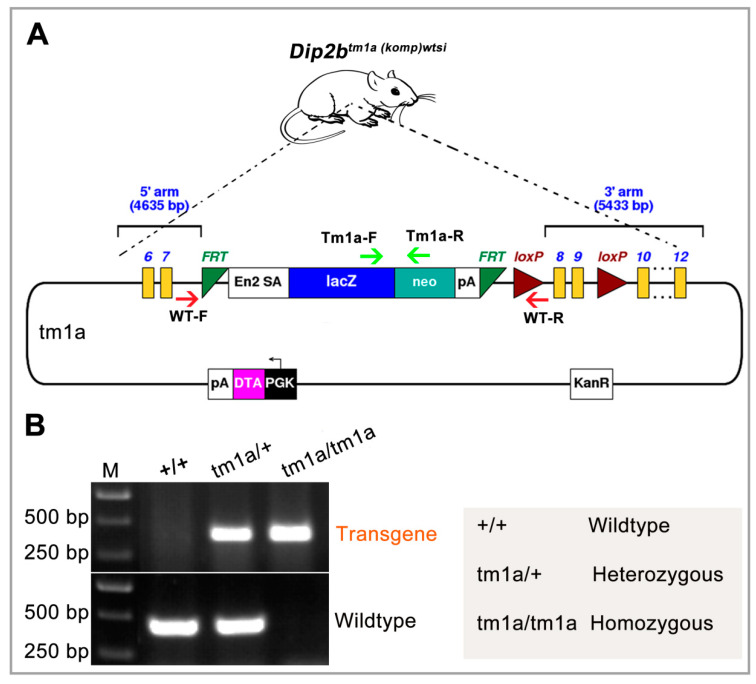
Structure of the *Dip2b^tm1a^* (KOMP) Wtsi allele: (**A**) Exons 8 and 9 are flanked by loxp while FRT, lacZ, neo, and FRT elements are inserted in intron 7. En2 SA sequences will trans-splice lacZ to exon 7 and be frame matched. LacZ-Neo-pA is a cDNA coding a fusion protein for both galactosidase as reporter and NEO expression as positive selection marker. PGK-DTA-pA is a negative selection marker for gene targeting in ES cells. KanR is a bacterial selection marker; (**B**) Genotyping by PCR. A 300 bp band appeared on gel for transgene allele and 350bp band for wild type allele. M, Molecular weight marker.

*Dip2b* transgenic mice containing the targeted *Dip2b^tm1a(komp)wtsi^* locus (*Dip2b^tm1a^*) were generated by insertion of a targeted trap tm1a-knockout-first allele [25], in which a promoterless selection cassette was inserted into the seventh intron of the *Dip2b* allele consisting of an internal ribosome entry site (En2), splice acceptor (SA) site, and a β-galactosidase (LacZ) reporter fused in frame to a promoterless neomycin resistance marker (Figure 1A). The presence of a splice acceptor site allows LacZ-Neo cassette to become part of exon 7, and En2 warrants internal translational start of LacZ-Neo cassette. Tm1a locus genotyping was performed using primers Tm1a-F and Tm1a-R to give a mutant specific band of 300 bp. The wild type primers WT-F and WT-R amplify the intron 7 region and give a product of 350 bp (Figure 1B).

The expression of *Dip2b* during embryogenesis was analyzed by taking advantage of LacZ gene expression from the *Dip2b* locus. The β-galactosidase activity of the LacZ gene was analyzed by X-gal staining of embryos of *Dip2b^tm1a/+^* × *Dip2b^tm1a/+^* mating. Gene expression was analyzed by qPCR as well. *Dip2b* mRNA was detected as early as in E6.5 embryos and increased from E8.5 thereafter.

**Figure 2:**
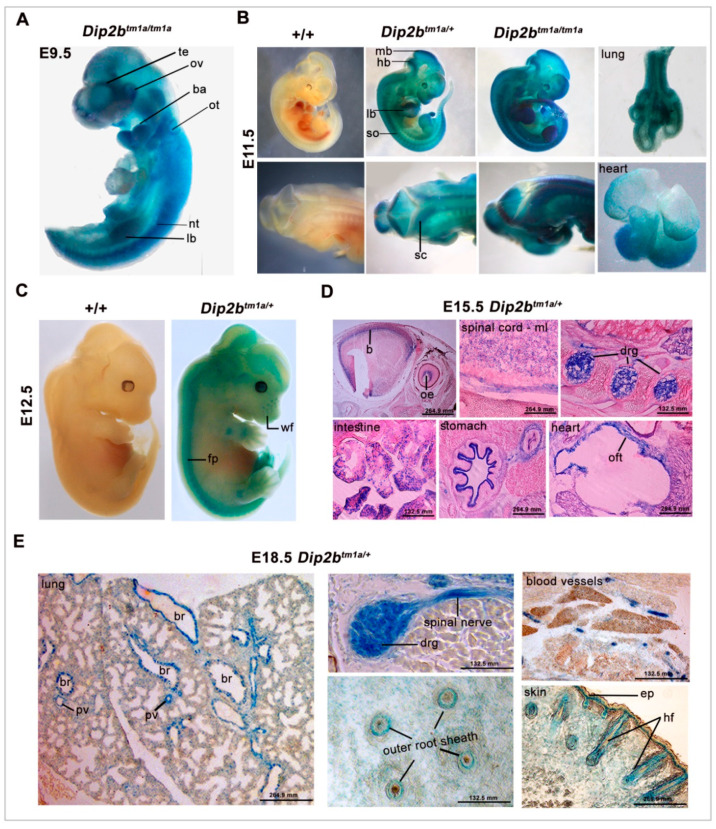
LacZ staining: (**A**) Whole mount of E9.5 *Dip2b^tm1a/+^* mice reveals lacZ expression in brain and dorsal tissues. Staining is intense at telencephalon (te), optic vesicle (ov), branchial arch (ba), limb bud (lb), and neural tube (nt); (**B**) At E11.5, *Dip2b* expression is concentrated in brain (mid brian, mb and hind brain, hb), dorsal neural tube (dsnt), intersomitic regions (so) and spinal cord (sc). At this stage, signals in heart and lung are also evident; (**C**) At E12.5, LacZ staining expands towards dorsal region specifically in brain (b), floor plate (fp) and whisker follicles (wf). Wild type littermates were devoid of lacZ staining; (**D**,**E**) Transverse sections of E15.5 and E18.5 *Dip2b^tm1a/+^* embryos were stained for LacZ and counter-stained with eosin. LacZ signals are shown in various neural tissues, in epithelial and endothelial cells of variety of organs. Olfactory epithelium (oe), mantle layer (ml), outflow tract (oft), blood vessels (bv), bronchus & bronchiole (br), pulmonary vessels (pv), epidermis (ep), and hair follicles (hf).

Whole Mount LacZ staining of E9.5 revealed a strong expression of *Dip2b* throughout the embryos (Figure 2A). Staining was detected in the neural tissues including neuroepithelium surrounding optical and otic vesicles, branchial arch, auditory pit, and limb buds. *Dip2b* expression was also visualized in the ventral region of the developing brain and neural tube at this stage of development. At E11.5, the intensity of β-galactosidase staining in the telencephalon, anterior midbrain, hindbrain, spinal cord, somite, dorsal neural tube, and the buds of developing limb was strongly increased as compared to E9.5 (Figure 2B). At this stage, an expression domain in lung and heart was evident. At E12.5, the LacZ expression pattern was similar to that of E11.5 with strong expression in developing whisker follicles of the whisker pad (Figure 2C). WT littermate (+/+) from all stages of development was devoid of the LacZ signal.

Staining of E15.5 and E18.5 embryo sections (18 μm) showed stronger and new expression domains compared to the early-stage embryos (Figure 2D,E). Expression was evenly distributed in the developing nervous system including the brain, spinal cord mantle layer, spinal nerves, and dorsal root ganglion. In heart sections, staining was located in the epicardium and outflow tract. Similarly, *Dip2b* was strongly expressed in epithelial-derived tissues, including intestine pseudostratified epithelium, stomach and skin epidermis, and hair follicles. In the lungs, *Dip2b* was expressed selectively in the respiratory epithelium lining of the intrapulmonary bronchus and bronchiolar duct. Staining of E18.5 frozen sections revealed that new domains of expression in the vascular systems are seen as in pulmonary vessels.

### 2.2. Dip2b Knockout Leads to Fetal Growth Restriction, Birth Weight Reduction and Perinatal Lethality

**Figure 3:**
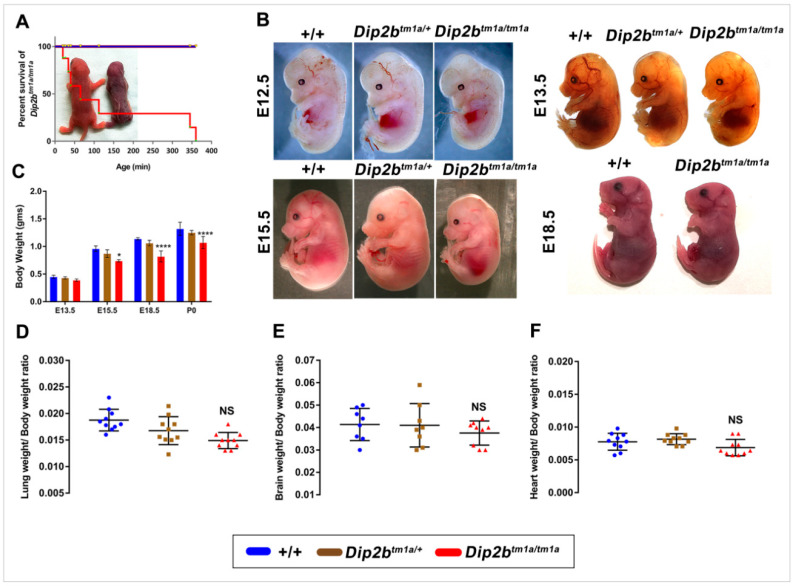
Phenotypical analysis: (**A**) Perinatal lethality in *Dip2b^tm1a/tm1a^* mice. Survival plot show significant lethality of *Dip2b^tm1a/tm1a^* mice at P0 (*p* < 0.0001, *n* = 36). Background picture shows cyanosis immediately after birth; (**B**) Representative images of newborns; (**C**) Body weight at E13.5—P0; (**D**–**F**) Average weights (in grams) of brain, lung and heart at E18.5. Wet weights of organs were normalized to total body weight. * *p* < 0.05, **** *p* < 0.0001. NS, not significant.

Observation of newborn pups from *Dip2b* heterozygous intercrosses demonstrated the lethality of homozygous *Dip2b^tm1a^* mice. From genotyping of 316 live pups from 36 litters, no *Dip2b^tm1a^* homozygotes survive to postnatal day 2 (Table 1). Further examination of 443 embryos of different prenatal stages (E9.5–18.5) showed expected Mendelian segregation of *Dip2b* genotype distributions. Hence, it is evident that Dip2b null mice survived embryonic stages but died after birth. To better estimate the time point of lethality, *Dip2b^tm1a/+^* intercross cages were kept on close observation for deliveries and deaths. Among the newborns, all the homozygotes died within 4–5 h after birth (Figure 3A). In summary, the Dip2b-tm1a allele is associated with postnatal lethality and all the homozygous fail to survive beyond p1.

To further characterize the developmental timeline of *Dip2b* mutant, litters derived from crosses of *Dip2b^tm1a/+^* heterozygous mice were examined at embryonic day (E) 11.5, E12.5, E13.5, E15.5, E18.5, and E19.5 (P0). No gross morphological defects were identified in *Dip2b^tm1a/tm1a^* embryos from E11.5 to E18.5. However, *Dip2b* homozygous embryos were comparatively smaller than wild type or heterozygous littermates (Figure 3B). Starting at E15.5, the *Dip2b^tm1a/tm1a^* mutant embryos showed a significant reduction in weight as compared to the weight of wild type or *Dip2b^tm1a/+^* embryos (Figure 3C).

### 2.3. Dip2b Inactivation Causes Respiratory Distress and Pathologic Lung Development

**Figure 4:**
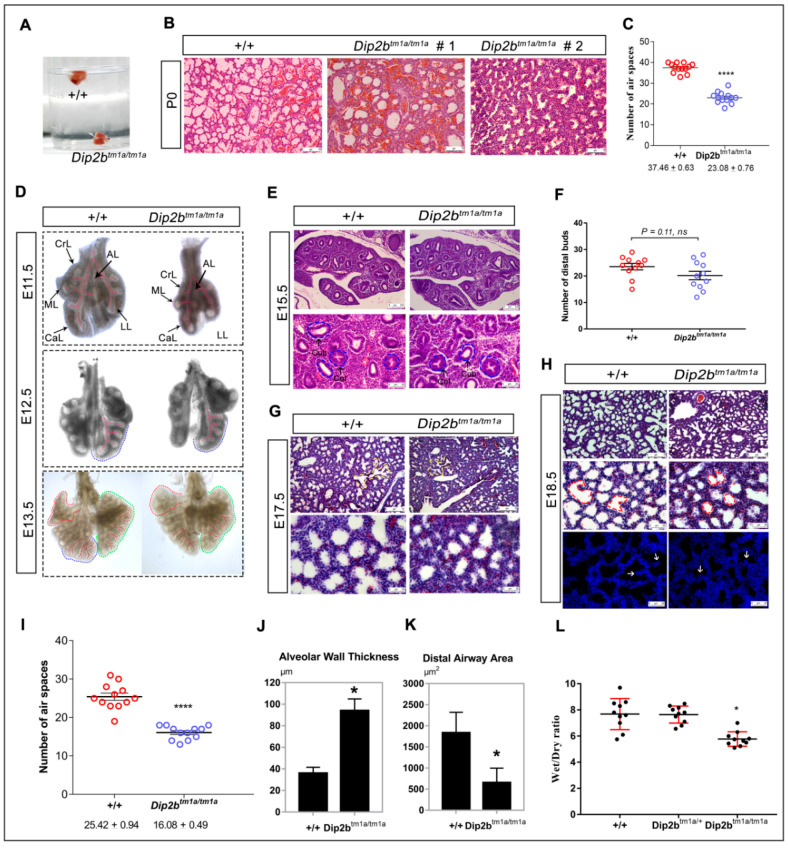
Lung structure comparison of WT and *Dip2b^tm1a/tm1a^* littermates: (**A**) Phenotype of a postnatal day 0 (P0) lungs after birth. The P0 lung was examined while it was floating in PBS; (**B**) The morphology of lung tissue and alveoli air space in WT and *Dip2b^tm1a/tm1a^* at P0. H&E-stained (Scale bar, 75 μm) lungs is shown; (**C**) Number of alveolar air space in WT and and *Dip2b^tm1a/tm1a^* mice; (**D**) Whole-mount of E11.5–E13.5 lungs of *Dip2b^tm1a/tm1a^* (left) and wild-type (right) mice. CrL, right cranial lobe; ML, right medial lobe; CaL, right caudal lobe; AL, right accessory lobe; LL, left lobe; (**E**) The morphology of lung tissue in WT and *Dip2b^tm1a/tm1a^* at E15.5. H&E-stained (Scale bar, 100 μm and 25 μm) lungs is shown. Col, columnar epithelium; Cub, cuboidal epithelium; (**F**) Quantification of distal epithelial buds in E15.5 lungs; (**G**,**H**) The morphology of lung tissue in WT and *Dip2b^tm1a/tm1a^* at E17.5 and E18.5 is shown. Alveolar septation is shown by white arrow. H&E and DAPI stained (Scale bar, 75 μm and 25 μm) lungs are shown; (**I**–**K**) Number of alveolar spaces, size and thickness in WT and *Dip2b^tm1a/tm1a^* mice at E18.5; (**L**) Wet/dry ratio of E18.5 lung tissues. Data are means ± SD (*n* = 3, * *p* < 0.5, **** *p* < 0.0001 and NS: *p* = 0.11) calculated by standard two-tailed unpaired *t*-test.

Direct observation of litters from *Dip2b^tm1a/+^* intercross revealed cyanosis in *Dip2b* homozygotes (Figure 3A), suggesting a lack of proper oxygenation due to circulatory or respiratory dysfunction. *Dip2b^tm1a/tm1a^* pups exhibited difficulty in breathing, characterized by a gasping mouth and exaggerated diaphragmatic contraction (Video S1). Therefore, lungs from wild type and *Dip2b^tm1a/tm1a^* were first investigated for architectural problems. Macroscopic examination showed that the gross morphology, the number of lobes, lung shape, and lung/body weight ratio were similar between *Dip2b^tm1a/tm1a^* and wild type mice at P0 (Figure 3D). No significant difference in the ratio of brain/body and heart/body weight between *Dip2b^tm1a/tm1a^* and wild type further confirmed that *Dip2b^tm1a/tm1a^* developed normally (Figure 3E,F). When intact lungs including trachea were placed in PBS and observed for flotation, lungs from wild type floated but *Dip2b^tm1a/tm1a^* sank, representing inadequate aeration (Figure 4A). It is clear that *Dip2b^tm1a/tm1a^* mice failed to fully inflate their lungs. Histology studies at P0 show that while wild type lung exhibited normal lung structure with inflated and expanded alveolar space, *Dip2b^tm1a/tm1a^* lungs displayed reduced alveolar sacs formation and unexpanded intra-alveolar septation. In more severe cases, *Dip2b^tm1a/tm1a^* lungs showed collapsed air sac formation with dense cellularity (Figure 4B,C). Based upon these phenotypic characterizations, we suspect that abnormal lung maturation is probably the primary cause of respiratory distress and death of *Dip2b^tm1a/tm1a^* neonates.

Anatomical and histological analysis of lungs at P0 suggested that homozygous *Dip2b^tm1a/tm1a^* mice were born with poor lung morphology. To investigate at what stage lung defects were first evident, we examined lungs from early developmental stages starting from branching morphogenesis to the saccular formation (E11.5–18.5). At E13.5, five lobes of right and left lungs were clearly distinguishable in both wild type and *Dip2b^tm1a/tm1a^* embryos. It was evident that *Dip2b^tm1a/tm1a^* lungs are comparatively smaller. Development of primitive bronchi that separate the lungs and development of epithelial buds that give rise to five lobes were visible but with a tendency to form less well-defined secondary and tertiary branches, as the pulmonary tree branching was extensive in wild type (Figure 4D). At E15.5, microscopically distinct columnar or cuboidal epithelium, correlating to the pseudo glandular period of lung development, is evident in both wild type and *Dip2b^tm1a/tm1a^* (Figure 4E). Although the total number of distal epithelial buds in *Dip2b^tm1a/tm1a^* lungs (20.18 ± 1.6, *n* = 11) was comparatively less than wild type lungs (23.55 ± 1.2, *n* = 11), the observed difference was not statistically significant (Figure 4F).

During the late canalicular stage of lung development at E17.5, wild type lungs displayed narrower terminal buds as expected. In contrast, *Dip2b^tm1a/tm1a^* lung had dense appearance, few distal saccular expansion and bronchus diversion (Figure 4G). At E18.5, wild type lung had many small sacs with thin septa, but in *Dip2b^tm1a/tm1a^* lung had markedly delayed septation (Figure 4H). In addition, the wild type lungs were highly branched and the wall of the saccules has multiple short buds that elongate to form secondary septa, whereas in *Dip2b^tm1a/tm1a^* lung, the buds were either short or completely absent. A quantitative analysis showed that on average, *Dip2b^tm1a/tm1a^* lungs had a small number of distal air sacs and increased thickness of alveolar wall in comparison to wild type lung at E18.5 (Figure 4I–K). In addition, Lung wet/dry ratio showed a reduced amount of lung fluid in *Dip2b^tm1a/tm1a^* as compared with wild type, presumably leading to the smaller alveolar space observed (Figure 4L). In particular, *Dip2b^tm1a/tm1a^* lung at E18.5 and P0 showed increased cellularity and reduced terminal air sacs formation. No other histological abnormalities in proximal airway epithelial, smooth muscle, or bronchial epithelial were observed from *Dip2b^tm1a/tm1a^* mice when compared between wild type. In addition, Sirius red staining did not show any significant differences in collagen deposition between wild type and *Dip2b^tm1a/tm1a^* at E18.5 (Figure S1). These results indicate that *Dip2B* is important for lung organogenesis and saccular stage lung development.

### 2.4. Dip2b Regulates Cell Proliferation but Not Apoptosis in Lungs

To evaluate if there was overall cell loss (Hypoplasia) in the lungs of *Dip2b^tm1a/tm1a^* mice, the left lung DNA content of the E18.5 embryo was accessed. While the bodyweight of *Dip2b^tm1a/tm1a^* was less than the wild type, there was a substantial increase in the total DNA in the mutant lung compared to wild type and heterozygous, with an increased ratio of DNA content to body weight (Table 2). Thus, while differentiation of type I and type II cells appears defective in the *Dip2b^tm1a/tm1a^* lungs (Figure 4, there is an apparent increase in cell number in the *Dip2b^tm1a/tm1a^* lungs, suggesting *Dip2B* may be involved in cell proliferation or apoptosis during lung maturation [26].

**Figure 5:**
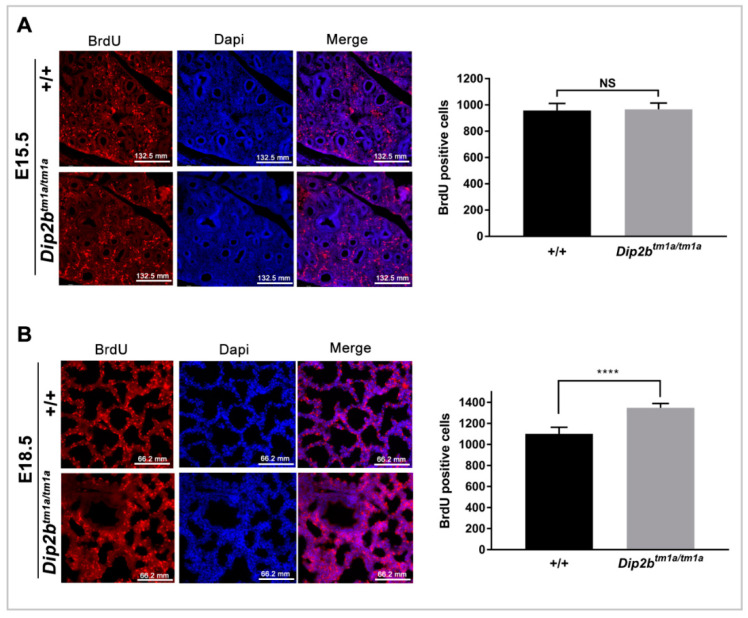
Cell proliferation assay by Bromodeoxyuridine (BrdU) incorporation in lungs: (**A**) BrdU-Positive cell number in E15.5 lungs. Scale bars, 132.5 μm; (**B**) Cell proliferation assay at E18.5. Scale bars, 66.2 μm. NS = not significant, **** *p* < 0.0001 by student’s *t*-test. Three pubs were analyzed per group.

To evaluate if cell proliferation was altered, E15.5 and E18.5 pregnant dams were injected with 5-bromo-2-deoxyuridine (BrdU). *Dip2b^tm1a/tm1a^* lungs at E18.5 showed a significant increase in BrdU positive cell compared to wild type lungs (1350 ± 10.82 versus 1102 ± 16.51, *n* = 14, *p* < 0.0001, Figure 5B), indicating that a higher level of cell proliferation has occurred in the *Dip2b^tm1a/tm1a^* lungs. This apparent increase in proliferation is consistent with the thickened mesenchyme and increased DNA content in *Dip2b^tm1a/tm1a^* lungs and may reflect a failure of the normal reduction in mesenchymal proliferation seen during lung maturation [26]. There was no difference in BrdU incorporation at E15.5 between *Dip2b^tm1a/tm1a^* and wild type (966.6 ± 13.81, *n* = 12 versus 957.6 ± 15.45, *n* = 0, *p* = 0.66; Figure 5A), suggesting that cell proliferation was intact at the branching stage but interrupted during the saccular stage and that defective proliferation is coupled to the maturation defect. Cell apoptosis was measured by terminal deoxynucleotidyl transferase dUTP nick-end labeling (TUNEL) assay. At E18.5, a comparable number of TUNEL positive cells were detected in both groups (1116 ± 19.7 versus 1102 ± 20.49, *n* = 15, *p* = 0.61, Figure S2).

### 2.5. Dip2b Regulates Expression of Cell Cycle Genes, Epithelial Cell Differentiation Markers and Lung Mediators

**Figure 6:**
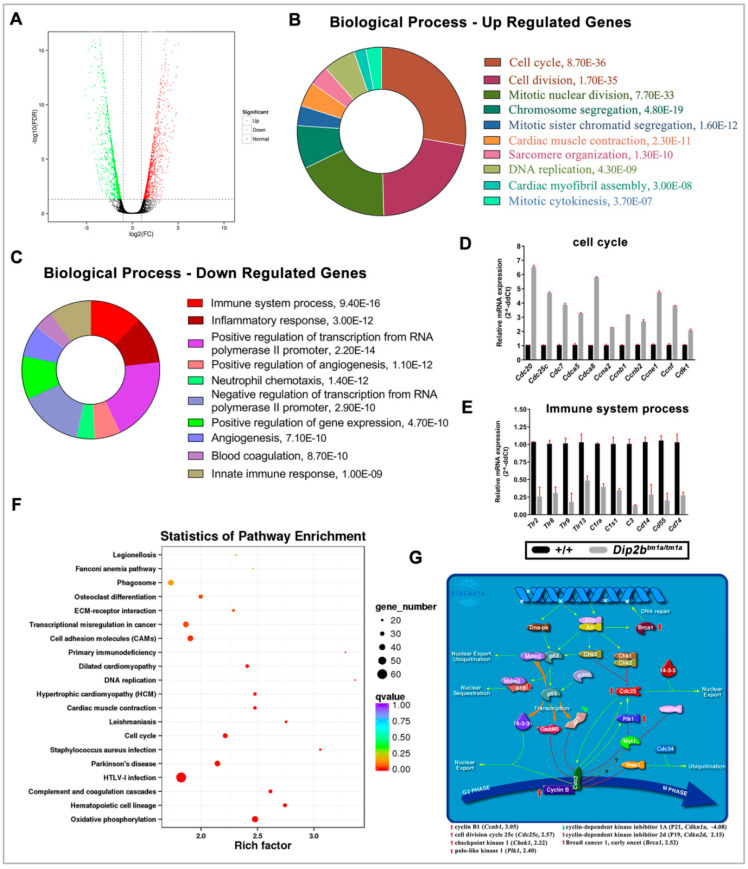
E18.5 Lung transcriptome analysis defined by FDR < 0.01 and Fold change > 2: (**A**) Volcano plots of differentially expressed genes (DEGs). The red and green dots represent up-regulated and downregulated genes respectively; (**B**–**E**) Gene ontology (GO) analysis of DEGs; (**B**,**C**) Pie charts of top 10 overrepresented biological process terms from upregulated and downregulated genes respectively; (**D**,**E**) The mRNA expression levels of 11 & 10 genes annotated under biological process term ‘Cell cycle’ and ‘immune system process’ determined by qPCR. The red error bars represents standard deviation; (**F**,**G**) Pathway enrichment analysis; (**F**) KEGG pathway enrichment scatter plot. Each circle represents a KEGG pathway. The *Y*-axis represents the name of the pathway and the *X*-axis indicates Enrichment Factor, indicating the proportion of the annotated genes in the pathway. The lowest Q value represents the most significant pathway; (**G**) Biocarta G2/M phase transition pathway. A total of seven DEGs were annotated. Red arrow represents upregulated genes while green arrow represents downregulated genes.

To further examine whether the loss of *Dip2b* alters lung gene expression profiles, we performed RNA sequencing of E18.5 lungs (Table S1). The study found 1431 genes differentially expressed (Fold change ≥ 2, FDR < 0.01), comprising 696 up and 735 downregulated in *Dip2b^tm1a/tm1a^* lungs (Figure 6A and Table S2).

Gene Ontology (GO) analysis was performed on these differentially expressed genes (DEGs) by separating upregulated from downregulated genes to isolate particular biological processes that may explain the distinct phenotype observed in E18.5 *Dip2b^tm1a/tm1a^* lungs. The top 10 enrichment terms for up and downregulated genes are listed in Figure 6B,C. Results show that 94 up-regulated genes are highly overrepresented under GO term cell cycle, including several cell division cycle (Cdc20, Cdc45, Cdc6, Cdc7, Cdca2, Cdca3, Cdca5, and Cdca8), cyclin (Ccna2, Ccnb1, Ccnb2, Ccne1, and Ccnf) and kinesin (Kif11, Kif18b, Kif20b, Kif23 and Kif2c) family member genes, all of which have well-established functions in cell cycle control (Table S3). The enrichment of cell proliferation was observed at E18.5 *Dip2b* lungs. Among GO terms from downregulated genes, 54 genes annotated under the immune system process are highly overrepresented (Table S3). The mRNA levels of these DEGs from the cell cycle and immune system process are confirmed by quantitative real-time PCR (QPCR) (Figure 6D,E). These results suggest Dip2B downregulates the cell cycle and upregulates the immune system during lung development.

To analyze whether these genes are over-presented on a pathway, KEGG analysis was performed. KEGG pathway analysis identified that oxidative phosphorylation and hematopoietic cell lineage pathways are significantly enriched in *Dip2b^tm1a/tm1a^* lungs (Figure 6F). Under the oxidative phosphorylation pathway, ATPase, NADH ubiquinone oxidoreductase, and cytochrome c oxidase family genes were upregulated, whereas mitochondria-encoded NADH ubiquinone oxidoreductase and ATP synthase membrane family genes were downregulated (Table S4). Similarly, genes annotated under hematopoietic cell lineage are mostly downregulated CD markers (Table S4). Biocarta pathway analysis shows a significant hit particularly on the G2/M phase transition pathway (Figure 6G). The decreased expression of cell cycle inhibitor Cdkn1a and increased expression of Ccnb1, Cdc25c, Chek1, Plk1, Cdkn2d, and Brca1 may have increased BrdU-positive cells in E18.5 *Dip2b^tm1a/tm1a^* lungs. These results indicate that Dip2B may regulate lung development through altering oxidative phosphorylation, hematopoietic cell lineage, and G2/M transition pathways.

**Figure 7:**
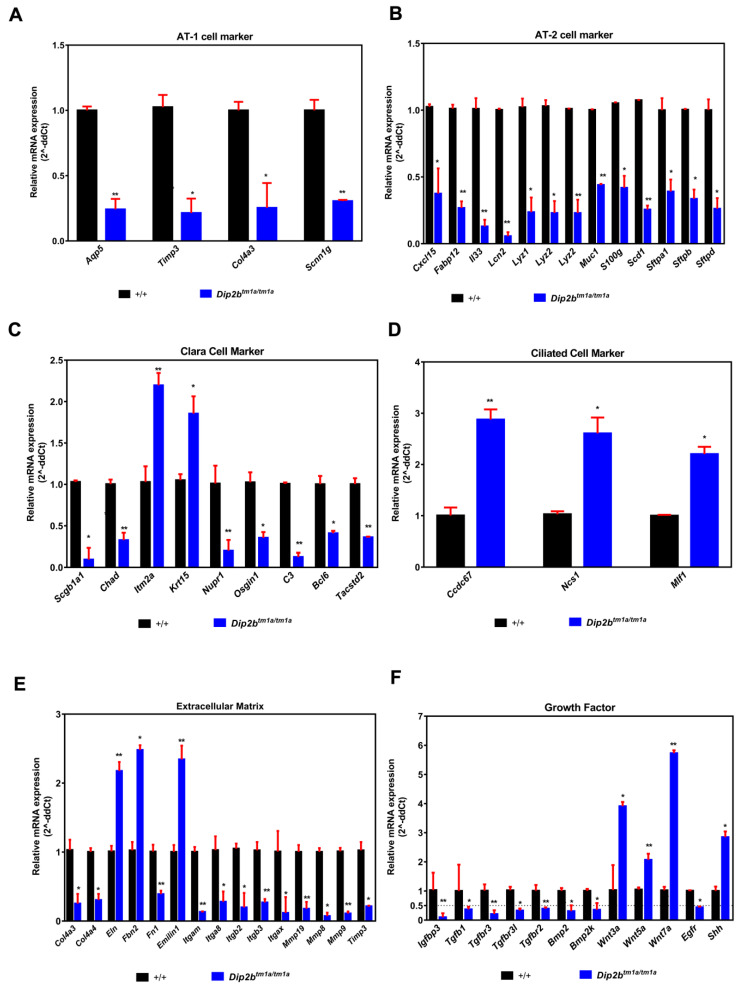
(**A**–**F**) Gene expression analysis of alveolar, bronchiolar cell markers and lung mediator genes by qPCR of E18.5 lungs. Mix of total RNAs from three pups were analyzed per group. Data are means ± SD (*n* = 3, * *p* < 0.05 and ** *p* < 0.001).

Lung morphogenesis at late gestation is characterized by a dramatic increase in epithelial cell differentiation markers [27]. To determine whether lung immaturity corresponds to reduced expression of these markers, we searched all the known markers related to alveolar and bronchial epithelial cell lineages in RNA sequencing data [28] and validated these markers by qPCR (Figure 7). Dip2b^tm1a/tm1a^ lung showed reduced transcripts of surfactant-secreting cuboidal alveolar type 2 (AT2) cell markers including Sftpa1, Sftpb, Sftpd, Cxcl15, Lyz1, Lyz2, Muc1, S100g, and Scd1, as well as flat alveolar type 1 (AT1) cell markers specialized for gas exchange including Aqp5, Timp3, Col4a3, and Scnn1g. Also, markers for Clara cell comprising Scgb1a1, Chad, Nupr1, Osgin1, C3, Bcl6, and Tacstd2 were also downregulated except for Itm2a, Krt15, which were upregulated. In contrast, markers of the ciliated cells including Ccdc67, Ncs1 and Mif1 were upregulated. Extracellular matrix and Growth factors mediate tissue interactions and regulate a variety of cellular functions that are critical for normal lung development and homeostasis [29,30]. In our study, we find several ECM like Col4a3, Col4a4, Itga8, Itgb2, Itgb3, Itgax, Mmp19, mmp8, and Mmp9 and growth factors like Tgfb family, Bmp family and Wnt family genes were de-regulated (Figure 7E,F). These results indicate that Dip2B regulates expression of terminal differentiation markers of Type1, Type 2 and markers of the ciliated cell along with ECM and growth factors critical for terminal lung development.

## 3. Discussion

In the present study, the functional roles of Dip2B were investigated using *Dip2b^tm1a/tm1a^* mice. *Dip2b^tm1a/tm1a^* mice are conditional null and LacZ reporter also generated by KOMP using tm1a knockout first strategy [25]. We have used heterozygous *Dip2b^tm1a/+^* for expression study and homozygous *Dip2b^tm1a/tm1a^* mice for functional analysis. Abundant LacZ expression has been found in most analyzed organs starting from the early stages of development and mapped to distinct cell types or individual cells. The prominent sites of expression were various neuronal, myocardial, endothelial, and epithelial cell types, indicating the potential biological role of Dip2B in these specific cells. The observed LacZ expression pattern partially overlaps with Dip2A, which is an indication of potential compensation roles in these tissues [22]. Mapping Dip2B expression patterns in embryonic tissue are of particular interest, since, until now, validation of in situ Dip2B expression analysis in mouse embryo has not yet been available. Also, the transcriptional mechanism behind the normal constitutive or cell-type-specific expression of *Dip2b* remains unknown. *Dip2b^tm1a^* mouse model provides a perfect tool to study Dip2B-regulated genes and pathways.

Despite over 15 years of history, the physiological role of the *Dip2b* gene in mammals is still poorly understood. In recent years, DIP2B has drawn increased attention for its potential role in neurocognitive disorder [15,17]. However, there has been no evidence either from tissue culture or animal models that show a contribution of Dip2B in organogenesis. In this study, we have discovered that mouse homozygous inactivation of Dip2B exerts intrauterine growth restriction and death of newborns within a few hours due to abnormal lung pathology and neonatal respiratory distress (NRD). *Dip2b^tm1a/tm1a^* mutants showed the apparent arrest of fetal lung maturation at the saccular and late canalicular stage as evidenced by morphology (saccular septal hyperplasia and reduced air sac formation) and decreased expression of epithelial differentiation markers. The invariable atelectasis is likely due to the failure in turning off cell proliferation, elevated cell cycle, and cell division signaling molecules. The present study, for the first time, reveals an indispensable physiological role of Dip2B in prenatal lung maturation and animal survival.

Neonatal respiratory distress syndrome (NRDS), often occurring in premature or low-weight infants, is associated with anatomical immaturity of the lung, increased compliance of chest wall, and inefficiency of premature pulmonary lymphatics coupled with decreased surfactant function or production [31,32,33]. Approximately 30% of all neonatal deaths result from complications of NRDS. The development of respiratory distress with gasping, cyanosis, and low birth weight in *Dip2b^tm1a/tm1a^* mice present a similar phenotype to the clinical manifestation of neonatal respiratory distress syndrome (NRDS). Given the decreased expression of *Sfpta1*, *Sfptb* and *Sftpd*, it is possible that *Dip2b* deletion might alter the activity of secreted surfactants, which is likely associated with respiratory failure at birth. Our findings may advance the knowledge of the underlying molecular mechanism of NRDS.

Prenatal lung maturation proceeds through a series of morphologically and biochemically defined stages [34,35]: from budding (E9.5) to initial development of bronchial and respiratory tree with the formation of an undifferentiated primordial system (Pseudoglandular from E9.5 to 16.6), to development of terminal sacs and vascularization (canalicular from E16.5 to 17.4), to increment in the number of terminal sacs and vascularization along with type I and II cells differentiation (Sacculation from E17.5 to P5). The final maturation of the terminal sacs into alveolar ducts and sacs (Alveolization) occurs postnatally from approximately P5 to P30 [36,37]. *Dip2b* mice form the correct number of lung lobes, together with the fact that all the *Dip2b* knockout mice survive embryogenesis, suggesting that *Dip2b* might not be a master gene that regulates the early stage of lung development. Instead, *Dip2b* might play a more important role during the mid-to-late stages of lung development. Our further evidence confirms that lung pathology induced in *Dip2b* mice is due to a delay in fetal pulmonary maturation rather than early-branching morphogenesis. Although we evidenced a delay in branching morphogenesis manifested at the early psuedoglandular stage (E11.5–E13.5), the overall growth of epithelial buds was comparatively comparable to wild type at the late psuedoglandular stage (E15.5). An increase in cell proliferation and cell apoptosis is involved in peripheral mesenchymal during normal branching morphogenesis [38]. The observation of a comparable number of BrdU positive cells between *Dip2b* and wild type lung at E15.5 supports the normal branching process. Nevertheless, Starting from E17.5 (the saccular stage), an obvious defect in alveolar maturation was seen in mutant lungs, as evident by a lack of saccules formation and thick interstitium septae although being grossly normal in architecture, collagen deposition, and intact pulmonary vessel development. Since the morbidity and mortality of premature infants are strongly associated with a failure of lung maturation [39], these mice may provide a useful model to investigate the later stages of prenatal lung maturation.

Our analysis showed that there is an apparent increase in the amount of DNA of *Dip2b^tm1a/tm1a^* lungs, suggesting disturbed cell proliferation and/or apoptosis during lung maturation. Increased number of proliferating cells in *Dip2b^tm1a/tm1a^* versus wild type as measured by BrdU staining and comparable cell death between wild type and mutant detected by TUNEL staining at E18.5 validated the outcome of a net increase of DNA content in mutants. Besides, these findings also indicate that the interstitium thickening of septae is the result of uncontrolled cell proliferation and not because of the failure of apoptosis. Previous studies of corticotropin-releasing hormone (CRH) and glucocorticoid (GR) knockout mice have shown similar findings in which hypercellularity, failure of septal thinning and immature airway formation were seen with an abnormally prolonged period of lung proliferation rather than the failure of timely apoptosis [40,41].

Lung maturation defect at late stages of fetal development has been seen in mice containing targeted disruption of genes encoding transforming growth factor [42], glucocorticoid receptor [39] neural precursor cell-expressed developmentally down-regulated 4 (NEDD4) [43], latent transforming growth factor β binding proteins [44], and other genes [45,46,47,48]. The molecular mechanism linking this diverse set of molecules to the development events essential for lung maturation has not yet been determined. As for the underlying molecular mechanism for the observed defects in our study, the RNA-sequencing-based gene expression analysis showed that the expression of over 1400 genes was affected by the loss of *Dip2b* in E18.5 lung, indicating that many factors including ECM and growth factors are likely responsible for the lung phenotype of *Dip2b^tm1a/tm1a^*. Given the increased cell proliferation in *Dip2b^tm1a/tm1a^* lungs, it is possible that upregulation of several genes annotated to biological processes such as cell proliferation, cell cycle, and cell division, results in the maturation arrest. Thus, *Dip2b* may be involved in either mesenchymal or epithelial cell exit from the cell proliferation, cell cycle, and cell division essential for normal maturation of the lung. As this is the first report on *Dip2b^tm1a/tm1a^*, further studies are still needed to validate this hypothesis. Future studies will also be needed to characterize the underlying mechanism of Dip2B in regulating biological process immune response and pathway oxidative phosphorylation.

During the alveogenesis process, epithelial progenitor cells differentiate into alveolar type 1 (AT1) and alveolar type 2 (AT2) cells [27]. The RNA-sequencing analysis and qPCR validation showed that structural immaturation of *Dip2b^tm1a/tm1a^* lung was accompanied by the decreased mRNA level of several AT1 and AT2 differentiation markers, indicating impaired epithelial cell differentiation. The notable reductions of these markers caused by *Dip2b* deletion at the protein level remain to be elucidated. Also, it will be important to determine the effect of the declining level of AT cell transcripts on cell maturation. However, our model is in agreement with many genetically immature lung phenotypes, such as Rcn3 [49] and Fstl [50], in which increased cell proliferation in the saccular septa is accompanied by inhibition of epithelial cell differentiation due to reduced AT1 and AT2 marker expression.

In the present study, we provide evidence that *Dip2b* may modulate the G2/M phase transition pathway. Our RNA-sequencing analysis showed that *Dip2b* deletion resulted in an upregulation of the *Ccnb1* gene, which is a regulatory subunit of mitosis promoting factor (MPF) needed for mitotic progression [51,52,53]. Cyclin B along with *Cdc2* activation promotes M-phase entry in the cell cycle. Several factors regulate *Ccnb:Cdc2* complex, which include *Cdk1* inhibitor *Cdkn1a* [54]. Similarly, *Cdc25C* is one of three *Cdc25* isoforms present in mammalian cells and, in combination with *Cdc25B*, promotes activation of MPF [54]. Drosophila polo kinase and its orthologues in other eukaryotes are also important regulators of G2/M transit, mitotic progression, cytokinesis, and exit from mitosis [55,56]. We hypothesize that downregulation in mRNA expression of *Cdkn1a* along with the upregulation of *Cdc25c* and *Polo1* may contribute to the ultimate activation of MPF and further promote M phase entry during the cell cycle. However, the ultimate function and mechanism of Dip2B in the G2/M phase transition require further exploration.

In summary, we provide evidence of an important role of Dip2B in late lung development. Insertion of Tm1a in the *Dip2b* allele allows us to study expression patterns of Dip2B expression and functional roles of Dip2B under inactivation. Knockout of Dip2B results in an intrauterine growth restriction and neonatal death possibly due to respiratory insufficiency. Delayed lung maturation includes increased lung tissue cellularity through cell proliferation, immature alveolar sacculation, and reduced alveolar septation. A decrease in mRNA levels for surfactant proteins and type I and type II epithelial cell markers provide the basis for the delayed maturation. In particular, gene expression profiling studies enabled us to demonstrate that Dip2B plays a novel role in the cell cycle, cell division, and G2/M phase transition. Results indicate that Dip2B is essential for lung development and survival.

## 4. Materials and Methods

### 4.1. Animals

*Dip2b* deficient (*Dip2b^tm1a(KOMP)Wtsi^*, abbreviated to *Dip2b^tm1a^* in this report) mice were purchased from KOMP (Mouse Gene Knockout Project). All animal experiments were conducted according to the guideline of the Institutional Animal Care and Use Committee, and Ethics Committee of Northeast Normal University (NENU/IACUC, 1 January 2018, AP2018011). Mice were housed in IVC cages in a clean facility of NENU under a 12:12 h light: dark cycles, 20 °C and 50 ± 20% humidity. Genotyping was performed by PCR and using two sets of primer: Tm1a-F (TGAGACTGAGCTTGGCTACCACA) and Tm1a-R (TCCTCCTACATAGTT GGCAGTGT), and WT-F (AGTTAAGGCTGAGCATGGTGGGA) and WT-R (TAGGGCTCTCACAGATCAGAGCT. Genomic DNA was extracted from tail tips by Proteinase K (Sigma) digestion [21]. Homozygotes *Dip2b^tm1a/tm1a^* offspring were generated by intercrossing of heterozygotes (*Dip2b^tm1a/+^*) on C57Bl/6 genetic background.

### 4.2. LacZ Staining

Visualization of beta-galactosidase (LacZ) expression was done in whole mount embryos or organs dissected from *Dip2b^tm1a/+^* pregnant dams. For whole mount staining, embryos from E9.5, E11.5 and E12.5 were harvested in ice-cold 1× PBS and fixed for 30 min in 2% PFA, 0.25% glutaraldehyde and 0.01% NP40 in PBS. Embryos were then washed three times, 15 min each in 2 mM MgCl_2_, 0.02% NP40 and 0.01% Na-deoxycholate in PBS, and then incubated at 37 °C overnight in 50–100 mL of an X-gal staining solution containing 30 mM K_3_Fe(CN)_6_, 30 mM K_4_Fe(CN)_6_·3H_2_O, 2 mM MgCl_2_, 0.01% Na-deoxycholate, 0.02% NP40, and 1 mg/mL 5-bromo-4-chloro-3-indolyl-β-D-galactopyranoside in PBS (pH 7.5). Embryos were post-fixed in 4% PFA overnight and stored in 70% ethanol at 4 °C. For frozen section staining, whole embryo and organs from E15.5 and E18.5 were dissected, fixed and washed as above. Samples were incubated in 20% sucrose overnight at 4 °C and then embedded in OCT compound (Tissue-Tek, Torrance, CA, USA) on dry ice. Sections were cut at 16 μm thickness and mounted on SuperfrostTM Plus microscope slides and allowed to dry for 30 min at room temperature, and staining was performed as above. Sections were post-fixed and counterstained (only E15.5) with 0.25% eosin solution. Wild type mice were used as a negative control. Images of whole mount embryos and frozen section tissues were taken using Olympus light microscope (SXL-ILLB-200, Tokyo, Japan) and Canon digital camera (DSI26431, Tokyo, Japan).

### 4.3. Cesarean Delivery and Weight Measurements

Timed mating was set up by crossing heterozygous *Dip2b^tm1a/+^* females with *Dip2b^tm1a/+^* males. Female mice were checked for copulation plugs and designated as embryonic development day E0.5 of gestation. All females were separated and housed individually after a vaginal plug was identified. Pregnant females were sacrificed at different stages of gestation, and embryos were harvested by cesarean section. Each embryo was weighed and immersed in 1× PBS and further subjected to morphological study. Embryos were then dissected under a stereomicroscope (Olympus SZX7, Tokyo, Japan). Whole embryos and individual organs were weighed using an analytical balance (Shenyang Longteng Electronics Co., Ltd. ESJ182, Shenyang, China).

### 4.4. Hydrostatic Lung Test

A non-recovery cesarean section of *Dip2b^tm1a/+^* intercrossed female dams was performed at E19.5 (6–8 h before birth). Embryos were collected and placed on a 37 °C heat pad and observed. After 60 min of observation, embryos were decapitated, and lung with trachea attached was placed into PBS and observed for floatation.

### 4.5. Weight Measurement of Lungs

The embryonic day (E) 18.5 lungs were removed from the chest cavity en bloc, dried with blotting paper and weighed to obtain wet lung weight. Lungs were then placed in a 60 °C oven overnight to obtain dry weight.

### 4.6. Lung Histology/Staining

E15.5 and E17.5 embryos were dissected from the uterus and fixed in 4% PFA for 24 h at 4 °C. The lungs from E18.5 embryos and P0 neonates were fixed for 24 h in 4% PFA at 4 °C. The fixed tissues were then washed twice in 1X PBS and stored in 70% ethanol at 4 °C. Briefly, tissues were dehydrated through a series of graded alcohols, cleared in xylene, infiltrated, and embedded in paraffin. Samples were taken from all five lobes. Six-μm-thick sections were stained with H&E. For Sirius red staining, paraffin section were hydrated and stained in sirius red solution (Sigma, Changchun, China) as instructed. Images were taken using Leica DMi8 light microscope (Leica Microsystem, Shanghai, China).

### 4.7. Analysis of Lung Branching Morphogenesis

Pregnant Dip2b^tm1a/+^ mice were sacrificed at E11.5, E12.5, E13.5, and E15.5 stages, and embryonic lung was dissected. Whole mount lungs from E11.5–E13.5 and paraffin-embedded lungs section from E15.5 were examined to verify the quantification of terminal branching. Branching morphogenesis was expressed as the number of epithelial buds visible around lung peripheries, *n* = 3.

### 4.8. Quantification of Lung Alveolar Sac Formation

Lung alveolar sac formation analysis was performed on H&E-stained sections at E18.5 and P0. The number of terminal respiratory air sacs was calculated using Adobe Photoshop 6.0 software. Multiple measurements were performed on randomly selected 66.2 m fields located at the distal part of the lung sections. The method for quantification of alveolar wall thickness was previously described [57]. The areas for distal airways were measured by using the area measurement function in ImageJ and calculated for mean value and SD. For each sample, three pictures were taken under a 40× objective lens. Three pubs from each group were analyzed.

### 4.9. 5-Bromo-2-Deoxy-Uridine (BrdU) Labeling

Pregnant dams at E15.5 and E18.5 were injected intraperitoneally (120 μg/gram body weight) with BrdU solution (Sigma Cat#B5002). Two hours later, mice were sacrificed and embryos were harvested. Lungs from all embryos were removed and embedded in OCT, sectioned in cryostat microtome, and processed for immunohistochemical evaluation [24]. BrdU was revealed using the Anti-BrdU antibody (Rat monoclonal, Abcam Cat#ab6326) for 1.5 h at room temperature. Slides were counterstained with DAPI (Invitrogen, Cat#3571). Images were taken using DMi8 S platform live cell microscope (Leica Microsystem, China). Slides were scored by counting BrdU-labeled cells under microscopic fields (20X), *n* = 3 for E15.5, *n* = 4 for E18.5.

### 4.10. TUNEL Assay

Apoptosis was detected and quantified in E18.5 lung paraffin sections using In Situ Cell Death Detection kit (Roche, Beijing, China) following the manufacturer’s guideline [44]. Briefly, sections were treated in xylene and dehydrated in a series of graded alcohol and finally rinsed in 1× PBS. Sections were treated with proteinase K before adding tunnel reaction mixture. Slides were counterstained with DAPI, and images were taken under DMi8 S platform live cell microscope (Leica Microsystem, Shanghai, China). Ten fields for each slide were photographed and TUNEL-positive cells were counted.

### 4.11. RNA-Sequencing (RNA-Seq)

RNA-seq of E18.5 lungs was carried out as described before (Sah et al., 2019) using Illumina HiseqTM 2500 (Biomarker, Beijing, China). A total of three pairs of RNA samples (3 WT and 3 *Dip2b^tm1a/tm1a^*) were sequenced. Bioinformatics analyses were performed using GOseq R packages [58], Kobas [59] and DAVID software version 6.8. Sequence reads from RNA Sequencing generated in this study are deposited in the NCBI SRA database.

### 4.12. Quantitative Real Time PCR (qPCR) Validation of RNA-Seq Results

Total RNA (1 μg) from E18.5 lungs of WT and *Dip2b^tm1a/tm1a^* was subjected to cDNA synthesis in a 20 uL reaction using primescriptTMII cDNA synthesis kit (Takara, Dalian, China). All qPCRs were performed using Thermocycler (Analytik Jena AG, Jena, Germany) and SYBR II premix (Takara, Dalian, China). All results were normalized to housekeeping gene 18S ribosomal RNA and relative quantification was calculated using comparative threshold cycle (2^−ΔΔCt^) values for three biological replicates.

### 4.13. Statistical Analysis

The data are expressed as the SEM ± SD. Statistical analyses were assessed using unpaired *t*-test, with statistical significance *p* < 0.05. χ2 analysis was done using the online calculation chi-square tool at http://www.quantpsy.org.

### 4.14. Database

NCBI (SRA: PRJNA647345, https://www.ncbi.nlm.nih.gov/sra/PRJNA647345)5.

## Figures and Tables

**Table 1:** Survival of Dip2b^tm1a/tm1a^ mice.

Age	+/+	*Dip2b^tm1a/+^*	*Dip2b^tm1a/tm1a^*	*χ*^2^ 10df	*p* Value
E9.5	7 (23.3%)	15 (50.0%)	8 (26.6%)	0.81	0.26
E11.5	12 (23.5)	25 (49.0%)	14 (27.5%)	0.71	0.99
E12.5	22 (25.5%)	40 (46.5%)	24 (27.9%)	0.56	0.14
E15.5	25(24.5%)	48 (47.0%)	29 (28.5)	0.37	0.26
E18.5	46 (26.2%)	90 (51.4%)	39 (22.2%)	0.35	0.71
P1	102 (32.2)	214 (67.8%)	0 (0%)	0.61	0
Expected	25%	50%	25%		

χ2 analysis progeny of *Dip2b^tm1a/+^* × *Dip2b^tm1a/+^* timed mating’s with 6 degrees of freedom. Percentage of observed embryos is presented in parenthesis. *p* is the statistical significance value.

**Table 2:** Body weight and DNA contents of the left lung.

Genotype	No. of Pups	Average Body wt (g)	Left Lung DNA/Total Body wt (μg/g)
**+/+**	7	1.14 ± 0.027	43.6 ± 3.93
***Dip2b^tm1a/+^***	13	1.06 ± 0.050	52.9 ± 5.08
***Dip2b^tm1a/tm1a^***	6	0.82 ± 0.108	63.1 ± 6.47

Data are means ± standard deviation.

## Supplementary Materials

Supplementary Materials can be found at https://www.mdpi.com/1422-0067/21/21/8223/s1. VideoS1. Respiratory Distress among *Dip2b^tm1a/tm1a^* mice soon after birth. Figure S1. Picro-sirius red stained sections of lung at E18.5. Figure S2. Cell apoptosis assay by TUNEL staining. A. Immunofluorescent TUNEL staining of E18.5 lungs. Scale bar: 66.2 μm. B. Quantitative analysis by counting of multiple sections from 3 independent samples per group. NS = not significant by student’s *t*-test. Table S1. Summary of RNA-Seq mapping results. The raw reads were mapped to the Mus Musculus genome (GRCm38) using TopHat2. Table S2. List of DEG (Differentially Expressed Gene) from RNA-Seq analysis results. Differentail expression analysis was carried out using DESeq2. Table S3. List of DEGs annotated under Gene Ontology (GO) terms cell cycle and immune system process. Table S4. List of DEGs annotated under KEGG Pathway oxidative phosphorylation and hematopoietic cell lineage pathways.

## Abbreviations

*Dip2a*	Disco Interacting protein 2 Homolog a
*Dip2b*	Disco Interacting protein 2 Homolog b
*Dip2c*	Disco Interacting protein 2 Homolog c
LacZ	β-Galactosidase
BrdU	5-Bromo-2-Deoxy-Uridine
GO	Gene Ontology
KEGG	Kyoto Encyclopedia of Genes and Genomes
