# Supplementary figures and images for: Targeted Disruption of Mouse Dip2B Leads to Abnormal Lung Development and Prenatal Lethality
Source: Int J Mol Sci. 2020 Nov 3;21(21):8223. doi: 10.3390/ijms21218223 (PMC7663123; doi:10.3390/ijms21218223)

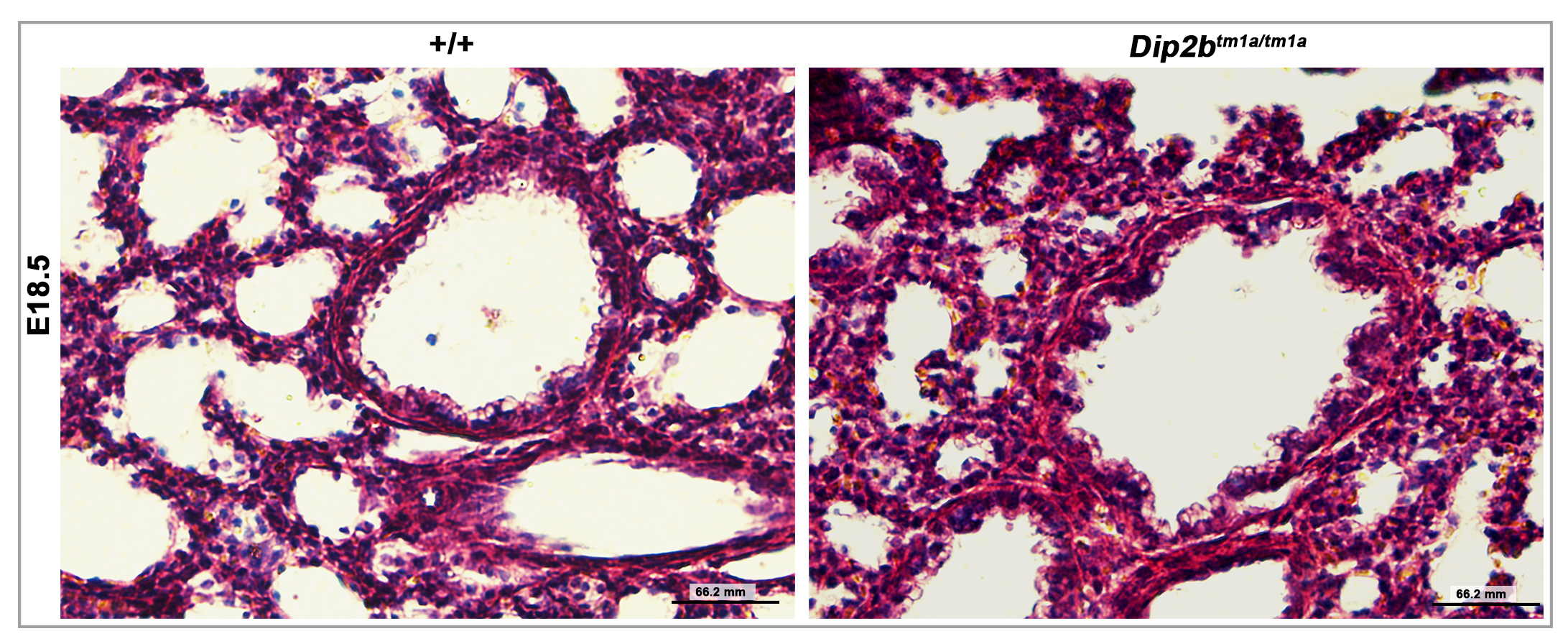

Supplement: Supplementary file 1 [file ijms-21-08223-s001.zip › ijms-958670 supplementary/Supplementary File/Figure S1.tif]

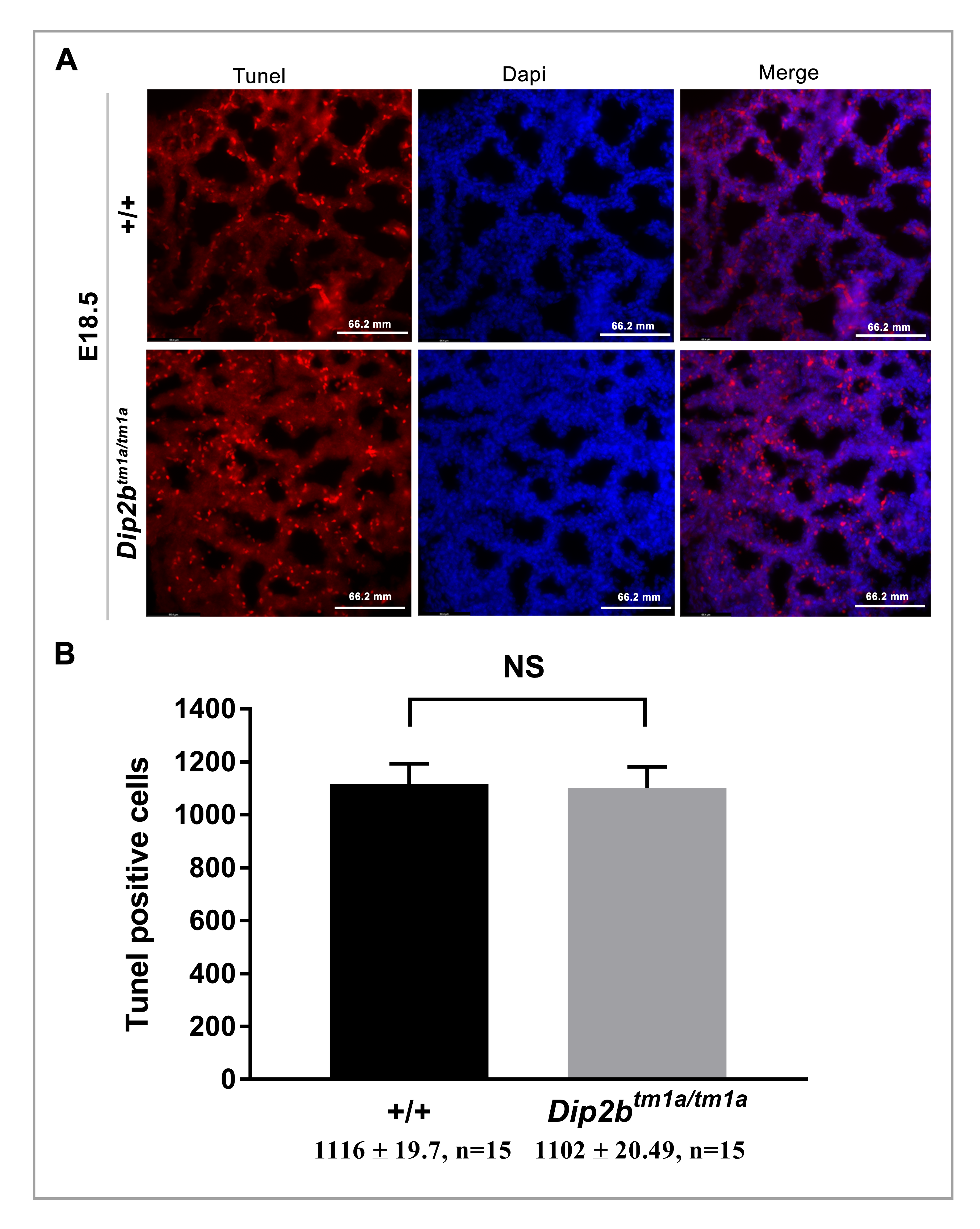

Supplement: Supplementary file 1 [file ijms-21-08223-s001.zip › ijms-958670 supplementary/Supplementary File/Figure S2.tif]
